# The Proper Treatment of Abortion: Reply to Dr. Smith’s Criticism

**Published:** 1897-04

**Authors:** W. J. Mathews

**Affiliations:** Austin, Texas


### Correspondence.

### The Proper Treatment of Abortion: Reply to Dr. Smith's Criticisms.

Austin, Texas, March 20, 1897.

*Editors Texas Medical Journal:*

In the February issue of your excellent Journal, Dr. Q. C. Smith criticised rather severely, and, I think, unjustly, certain improptu statements which I made before a late meeting of Travis County Medical Society. His criticisms are not only unjust, but in some particulars untrue.

Now, in order to correct any false impressions made upon your many cultured and intelligent readers, I shall be obliged, first, to define what I conceive to be the modern and scientific treatment of cases of abortion, which have gone beyond the reach of preventive measures; and secondly, to reply to his criticism.

Given, then, a case where there is no prospect of checking the progress of the abortion, and the expulsion of the ovum is inevitable, what, under such circumstances, is the best and safest plan of treatment to adopt? Where the os is not dilated, and yet there is considerable hemorrhage, we have been taught to use the vaginal tampon. That the tampon will effectually control hemorrhage no one doubts, but it cannot prevent the development of septicæmia, but may even favor its occurrence. No tampon can remain long in the vagina without acquiring an offensive odor. It generates septic matter, which may find its way through the cervix into the uterine cavity, and cause decomposition of the ovum. Just how soon a tampon may generate septic matter, none can tell, but all will agree that sooner or later it will do so. Therefore it cannot be recommended as an entirely safe method of treatment. The tampon certainly favors uterine contraction and the expulsion of the ovum. In days gone by I have, like others, used the tampon, and often successfully, but now, I think, we have other and safer methods in dealing with cases of this kind. The great and distinguished gynecologist, Lawson Tait, in his late well-known work on Diseases of Women, page 125, says: "No set of circumstances can justify plugging the vagina, except the direst emergency. It is a barbarous, slovenly, unscientific proceeding, and is generally based on incompetence, and instigated by terror." In a recent number of the New York *Gynecological Journal*, Dr. Grandin, one of the foremost obstetricians of America, says: "The old fashioned practice of putting tampons in the vagina, and waiting for nature, was still in force in New York City, and the old fashioned fear of certifying to the fact that the uterus was empty, still existed. It was time that tampons were relegated to the past, and that the fear of the curette, or finger being carried to the fundus and doing harm, was also relegated to the past."

In the *American Gynecological Journal* of January, 1896, J. Riddle Goffe, Professor of Gynæcology in the New York Polyclinic, after eulogizing the sharp curette as a life-saving instrument in abortions where the retained products of conception are septic, says: "In these cases it is not necessary to wait until the uterus has become septic, but in all cases of miscarriage, it is my custom, first, to explore the interior of the uterus, to make sure that it is absolutely empty of all products of conception, to cleanse it thoroughly, and to pack it with iodoform gauze. It is my firm conviction that where this treatment is applied to all cases of miscarriage, it is an absolute safeguard against further trouble."

I must confess that I have never tamponed a woman who was aborting, when the secundines still remained within the uterus, without feeling that I had not done my whole duty to my patient; nor have I ever left a patient in such a condition, feeling safe or happy as to the outcome of the case. Furthermore, the tampon will, in many cases, fail to cause the uterus to expel its contents, and then, under perhaps less favorable circumstances, we will be forced to dilate the cervix, and empty the uterus.

For many years I have followed the teaching of Professor Mundie, of New York City, viz., to empty the uterus immediately, of every woman who is aborting. In Cazaux and Tarnier's work on obstetrics, page 576, Professor Mundie says: "It has always been my practice to remove the whole ovum, fœtus and placenta, membranes and all, before leaving a patient who is aborting. To remove the placenta and membranes, I have used preferably my fingers; and when I failed with this method, I have always succeeded in detaching the secundines with a large blunt curette. My reason for this has been, that no woman has seemed to me free from the danger of hemorrhage or sepsis, as long as a portion of the secundines remained in the uterus; and I have never had occasion to regret following this practice." Professor Grandin, in the same journal, already referred to, says: "In my opinion there is only one way of treating abortions. When an abortion is found to be inevitable, the patient should be anæsthized, the field of the operation made aseptic, the uterus dilated, and everything removed from it. After inserting the finger, to be sure that everything had been expelled, the uterus should be drained with gauze. He wished, therefore, to enter a plea for the election of the surgical treatment of abortion, instead of the old fashioned method of waiting on nature, when she is often unable to satisfy the demands of modern obstetrics, to say nothing of modern surgery."

There can be no doubt that when an abortion is inevitable, the safest course to pursue is to empty the uterus at once; nor is this difficult to accomplish. In nearly every case where there has been much hemorrhage, the os-uteri is generally dilated, or, at least, soft and dilatable, so that sufficient dilatation can be easily accomplished with strong steal dilators, such as Goodell's or Ellinger's. This accomplished, the uterus can be quickly and safely emptied, when possible, with the finger, but if not, with a curette. If the curette is used, the finger should be introduced from time to time, to feel if anything remains in the uterus. Indeed, it is an excellent plan to keep a finger in the uterus while curetting. After curettement, the uterus should be irrigated or mopped out, so as to be sure that any remaining debris shall be removed. The next step is to pack the uterus tightly with iodoform gauze. This procedure will arrest any hemorrhage, which sometimes is quite profuse, after curetting. In a few minutes, or, as soon as all oozing ceases, the gauze is withdrawn, and another strip of dry iodoform gauze is pushed up to the fundus, and allowed to remain there, in order to insure good drainage. The vagina should also be loosely packed with iodoform gauze. Finally, Garrigues' antiseptic pad should be applied to the vulva, and retained in position with a "T" bandage. This line of treatment gives the physician complete control of his patient, and effectually prevents the meddling of ignorant, untrained nurses in the after-treatment of the case. The gauze need not be entirely removed from the uterus for four or five days, and must always be done by the physician himself. It goes without saying that everything coming into contact with a woman's genitals should be surgically clean. In fact all the antiseptic precautions employed in a vaginal hysterectomy should obtain here. In septic cases, the above treatment should be tried, no matter how seemingly hopeless the case may be. Here the gauze should not be left so long in the uterus, and the cleansings should be more frequent.

Now, I shall briefly reply to the doctor's criticisms, in the order in which he has written them.

First, my learned critic condemns the use of a sharp curette, especially in septic cases, fearing that the traumatism inflicted by the curette might open up the door of the absorbants, and permit the egress of enough ptomanic poison to kill the patient. Now, let us hear what the great masters say:

In a recent published work on gynæcology, by Keating & Co., the following language is used: "In septic inflammation following abortion, the uterine cavity should be freely curetted, the sharp curette being employed. The sharp curette is to be preferred at all times to the dull, for the same reason that a sharp knife is preferable to a dull one. A minimum amount of pressure accomplishes our purpose here, if a sharp curette is used. A maximum amount is needed with a dull instrument, and such pressure is far more likely to drive such an instrument through the softened uterine wall than the force requisite with a sharp instrument."

In another recent work, the American Text Book of Gynæcology, at page 225, the following declaration is made: "The dull curette scrapes off only the epithelial and softer external portions of the mucosa. Thus, its use may be harmful: for if a septic affection be local, and the epithelium of the rest of the organ has sufficient resistant power against the *cocci*, the procedure but removes the sole protection against a general affection, without going sufficiently deep to remove the *cocci:* and thus creates for the germs a new field for extension. So it is manifest that, in septic cases at least, the fancied safety of the dull curette, apart from its inefficiency, is a delusion."

On page 722 of the same work, Garrigues says: "Many obstetricians are opposed to the use of the curette in obstetric cases, maintaining as an argument, that new wounds are produced by it. In the writer's experience the curette is of great value, nay indispensible in abortion cases."

In the same work, page 874, Cameron, describing the treatment of septic cases, says: "It has been urged as an objection to those operators that the brush and curette denude the uterine walls, and open up fresh avenues for infection. Experience proves that such objections are groundless."

But to show the utter absurdity of the position taken by my learned friend, I shall relate the following case: Some time ago I was called to see a woman who, her husband said, had aborted about ten days previous to my visit. Patient said she had severe chills and high fever for four days. Her pulse was rapid and weak, and altogether her condition was very unpromising. I examined her, and found a piece of placenta firmly attached to the fundus of the uterus. The odor in this case was fearful. I tried to scrape away, with my finger nail, the tightly adhering piece of placenta, but failing in this I tried a blunt curette, with which I also failed. I then used a large sharp curette, Gill Wiley's, with which, in a few minutes, I scraped off the decomposing piece of placenta, and cleaned the uterus thoroughly. Before curetting, her temperature was 105° F. Twelve hours after, it was normal, and remained so. The only after-treatment was drainage with iodoform gauze. The retained piece of placenta was, in this case, the focus of infection, and to allow it to remain would have meant the woman's death. Now, according to my learned brother's teaching, it would have been wrong to scrape away the septic mass, which could only be accomplished with a sharp curette; because, by so doing, we would make a fresh wound, through which ptomanic poison would enter the circulation or lymphatic system and kill the woman. Would he, then, in his dread of exciting the hostility of the microbes, refuse to use the sharp curette and allow the unfortunate woman to die, with a piece of septic placenta within her uterus? Such would be a fair inference from his own statement.

Second.—He condemns carbolic acid as an antiseptic, because of its poisonous qualities. All I said to the society was, that after curetting and irrigating in septic cases, I was in the habit of mopping the endometrium with equal parts of carbolic acid and glycerine. After which I always filled the uterus with gauze, in order to absorb any surplus acid within the uterus; that in a few minutes the gauze was removed, and a fresh dry piece carried up to the fundus, to insure drainage. I also said that when I found any diphtheritic patches in the vagina, these were also mopped with equal parts of carbolic acid and glycerine. My object in mopping these parts was, both to kill the microbes in and around these vaginal patches, and to seal up the lymphatics and veins leading from such ulcers to the deeper parts. In accomplishing this, some use chloride of zinc and many other powerful escharotics, but none would be so simple as to use tincture of iodine.

You, Mr. Editor, seemed to think that no sane man would apply pure carbolic acid to the endometrium. Now, even "Tap Water" Tait would do this dreadful thing, and recommends it in his recent book on Diseases of Women and Abdominal Surgery, page 126. Probably you would dread absorption of the carbolic acid and systemic poisoning. Carbolic acid has the power of coagulating the albumen of the tissues. Applied to a recently curetted womb, carbolic acid immediately coagulates the albumen in the raw surface of the uterine walls, and thus effectually prevents its absorption. A very popular and effective treatment of hydrocele, after tapping, is to inject the *tunica vaginalis* with one fluid drachm of pure carbolic acid, and yet no case of poisoning has ever been reported. In removal of the uterine appendages the cut ends of the Fallopan tubes are often mopped with carbolic acid and dropped back into the peritoneal cavity with impunity.

Third.—Dr. Smith does not believe in tamponing the uterus when its cavity is surgically clean. Here my critic may be right. When there is good dilation of the cervix, and the uterine cavity is in an asceptic condition, gauze drainage might be safely dispensed with. Personally, I prefer to pack the uterus with iodoform gauze, whether it be septic or not, for the following reasons: As a general rule, the patient has lost a considerable amount of blood before she comes under the care of her physician. After the uterus has been emptied and curetted, there is often considerable bleeding, which is effectually checked by tightly packing it with gauze. Again, no matter how carefully we curette, whether in septic or non-septic cases, small particles of placental tissues or deciduous membrane may remain in the uterus; and nothing can so effectually prevent their decomposition, or the growth of bacteria as dry iodoform gauze packing. Besides, it insures a thorough aseptic condition of the uterus, promotes uterine contraction, and hastens the process of involution. Then, again, it gives the physician complete control of his case. Each day he must visit his patient, in order to remove a part of the gauze from the uterus, so as to permit gradual reduction in the size of the uterus. At each visit, after withdrawing a part of the gauze, he mops out the vagina so as to keep it as aseptic as possible. He also gives his personal attention to the external antiseptic pad. Thus, no one but the physician coming into contact with the case, he alone is responsible for the welfare of the patient. It will be claimed that the so-called busy physician has not the time to go through this daily routine of work. Then let him turn the case over to some conscientious physician, who is willing to spend a little extra time with such a case, especially when that time is spent in saving human life. Abortion cases thus treated should never develop septic fever, if the patient is not already septic before the institution of such treatment, and many septic cases, that would otherwise be doomed, can be saved.

His fourth arraignment of me is so manifestly ludicrous as scarcely to deserve serious consideration. The exact language I used before the Travis County Medical Society I do not now remember. The thought I meant to convey was, that if a physician were called to take charge of a woman who was aborting, who had healthy uterine appendages, and whose genital organs were not infected, and should such a woman develop severe septic fever during his management of the case, it would be but just to infer that there must have been something wrong in his technique or management of the case, and that, therefore, he would not be free from blame. Believing, as I do, that auto-infection is impossible, and that all pathogenic germs are introduced from without, it seems to me that it is the physician's duty to prevent the introduction of these germs, and thereby hinder the development of septic processes in the woman's genital tract. That this can be done has been abundantly proved by the records of our maternity hospitals, and in the private practice of a number of our well-known obstetricians. The Boston Laying-in Hospital in 1891 recorded 515 confinements without one death from septic infection. In the Sloan maternity, in New York City, there has been but one septic death in three thousand deliveries. In the New York Maternity Hospital there have been 957 deliveries without a death from sepsis. Equally good results are said to have been obtained by some of our best accouchers in private practice. Now, if these splendid results are possible in the management of labor at full term, why can we not obtain as good results in the management of abortions. In labor at full term there are lacerations and contusion of tissues from pressure, which are inviting points for infection. In abortions traumatisms are rare. The uterine absorbant surfaces are smaller, and the whole management of the case is much more under our control than in labor at full term, therefore, I think that we should have better results. At any rate, the past treatment of abortion has been very unsatisfactory. The ablest gynæcologists in this country believe that tubal diseases and pelvic peritonitis are oftener produced by the improper treatment of abortions than by any other cause. Therefore, my desire to urge a method of treatment, which I firmly believe will give infinitely better results than the older method. I am satisfied that if the treatment outlined in this paper should be adopted in every case of abortion, septic troubles would seldom or never occur. I am glad to be able to record the fact that the treatment of immediately emptying the uterus in cases of abortion is endorsed and practiced by one who, more than any other man in this country, led to victory the cause of antiseptic midwifery, Henry M. Garriques, M. D., Professor of Obstetrics in the New York Post-Graduate Medical School and Hospital. W. J. Mathews, M. D.

